# Supplementary material for: Visceral and subcutaneous abdominal fat is associated with non-alcoholic fatty liver disease while augmenting Metabolic Syndrome’s effect on non-alcoholic fatty liver disease: A cross-sectional study of NHANES 2017–2018
Source: PLoS One. 2024 Feb 23;19(2):e0298662. doi: 10.1371/journal.pone.0298662 (PMC10889905; doi:10.1371/journal.pone.0298662)
Supplement: S1 Fig — (PDF) [file pone.0298662.s002.pdf]

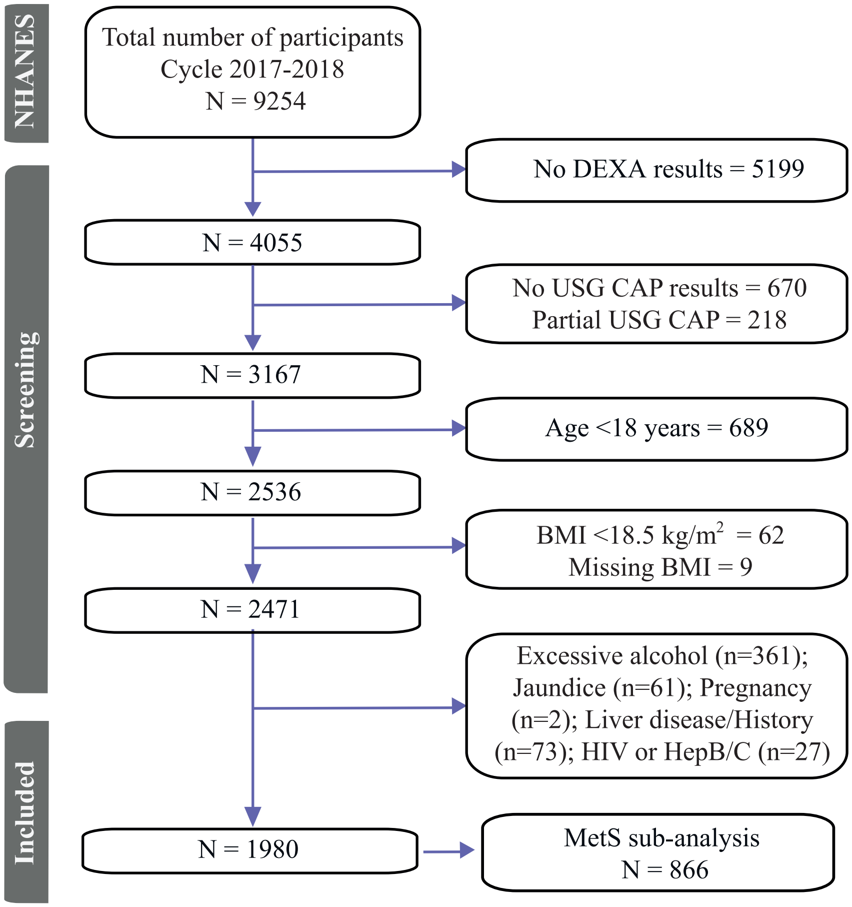

S1 Fig. Flow chart of the screening process for the selection of eligible participants to assess the effect types of abdominal adipose tissue have on the development of Non-Alcoholic Fatty Liver Disease.

Abbreviations: BMI: body-mass index; DEXA: Dual-Energy X-Ray Absorptiometry; HepB/C: Hepatitis B or C; HIV: Human Immunodeficiency Virus; NHANES: National Health and Nutrition Examination Survey; USG CAP: Ultrasound Controlled Attenuation Parameter.
