# Supplementary material for: Visceral and subcutaneous abdominal fat is associated with non-alcoholic fatty liver disease while augmenting Metabolic Syndrome’s effect on non-alcoholic fatty liver disease: A cross-sectional study of NHANES 2017–2018
Source: PLoS One. 2024 Feb 23;19(2):e0298662. doi: 10.1371/journal.pone.0298662 (PMC10889905; doi:10.1371/journal.pone.0298662)
Supplement: S1 Table — (PDF) [file pone.0298662.s003.pdf]

S1 Table. Comparison of Pearson correlation coefficients for TAFA, VAT, SAT, and BMI with liver steatosis, as measured with hepatic ultrasound with the Controlled Attenuation Parameter [a]

Vs.	TAFA	VAT	SAT
TAFA (r=0.569, p<0.001)	1.0	-	-
VAT (r=0.645, p<0.001)	Z=6.056, p<0.001	1.0	-
SAT (r=0.479, p<0.001)	Z=21.331, p<0.001	Z=10.146, p<0.001	1.0
BMI (r=0.580, p<0.001)	Z=1.303, p=0.190	Z=4.905 p<0.001	Z=10.064 p<0.001

Abbreviations: BMI: Body-mass index; SAT: subcutaneous adipose tissue; TAFA: total abdominal fat area; and VAT: visceral adipose tissue.

[a] Steiger's Z was calculated to compare between two correlations. Z-score >1.96 was considered significant.
