# Supplementary material for: Visceral and subcutaneous abdominal fat is associated with non-alcoholic fatty liver disease while augmenting Metabolic Syndrome’s effect on non-alcoholic fatty liver disease: A cross-sectional study of NHANES 2017–2018
Source: PLoS One. 2024 Feb 23;19(2):e0298662. doi: 10.1371/journal.pone.0298662 (PMC10889905; doi:10.1371/journal.pone.0298662)
Supplement: S2 Table — (PDF) [file pone.0298662.s004.pdf]

S2 Table. Predictability of type of abdominal fat depots and BMI for NAFLD as determined using receiver operating characteristic curve analysis.

Central obesity index	AUC [a]	vs VAT	vs SAT	vs BMI
TAFA (cm^2)	0.78 (0.76-0.80), <0.001	Z=5.13, p<0.001	Z=14.86, p<0.001	Z=2.74, p=0.006
VAT (cm^2)	0.83 (0.81-0.85), <0.001	N/A	Z=8.03, p<0.001	Z=2.92, p=0.003
SAT (cm^2)	0.74 (0.72-0.76), <0.001	Z=8.03, p<0.001	N/A	Z=8.73, p<0.001
BMI (kg/m^2)	0.80 (0.78-0.82), <0.001	Z=2.92, p=0.003	Z=8.73, p<0.001	N/A

Abbreviations: AUC: area under the receiver operating characteristic curve; BMI: body mass index; N/A: not applicable; NAFLD: non-alcoholic fatty liver disease; SAT: subcutaneous adipose tissue; TAFA: total abdominal fat area; and VAT: visceral adipose tissue.

[a] The number of cases with and without NAFLD was 34,633,354 weighted (725 unweighted) and 61,649,555 weighted (1255 unweighted), respectively.
